# New Appliances and Things Medical

**Published:** 1898-07-09

**Authors:** 


### NEW APPLIANCES AND THINGS MEDICAL.

[We shall be glad to receive, at our Office, 28 & 29, Southampton Street, Strand, London, W.C., from the manufacturers, specimens of all new preparations and appliances which may be brought out from time to time.]

### VIMBOS FLUID BEEF.

(Vimbos, Limited, 130, Queen Victoria Street, London, E.C.)

The fluid beef which is known under the above title is undoubtedly a highly concentrated extract of meat, differing, however, from many popular preparations in that it contains, in addition to the usual extractives, a large percentage of the nourishing elements of the meat from which it is prepared. In other words, Vimbos is not only a stimulant, it is a food, and both these valuable qualities are present in readily assimilable form. Whether taken simply diluted with water or as an accessory to cooking in the kitchen Vimbos will be found of great value, and from a physiological as well as from a pecuniary point of view it is a highly economical preparation.

### PURE CONCENTRATED SOLUBLE COCOA.

(J. S. Fry and Sons, Bristol and London.)

We have lately examined a sample of the above cocoa, and our present observations entirely coincide with our past experience of this excellent preparation. Its degree of solubility is noticeably high, the flavour is excellent, and for freedom from impurities it is beyond reproach. As a highly nutritive and digestible food we recommend it to the notice of our readers.

### ROYAL COFFEE.

(Messrs. Payton and Co., Limited, Tower Hill Warehouse, London, E.C.)

We have received two samples of ground coffee from the above firm; one a mixture with chicory at 1s. 4d. per lb., and the other pure coffee at 1s. 8d. Both are supplied in tins with hermetically self-sealing lids. The flavour and aroma is thus preserved for almost an indefinite period. We find both varieties most excellent of their kind, and as far as we have been able to examine them, they contain no impurities or admixture otherwise than that stated on the tins. For excellence of flavour and general convenience we recommend it to the notice of our readers.

With reference to our paragraph in last week's issue *re* the Valtine Meat Globules, we are asked to state that these are the invention of Mr. Charles R. Valentine.

